# A study on the characteristics of plasma polymer thin film with controlled nitrogen flow rate

**DOI:** 10.1186/1556-276X-7-62

**Published:** 2012-01-05

**Authors:** Sang-Jin Cho, Jin-Hyo Boo

**Affiliations:** 1 Department of Chemistry and Institute of Basic Science, Sungkyunkwan University, Suwon 440-746, South Korea

**Keywords:** PECVD, plasma polymer, N-ThioPP, optical, physical, and chemical properties

## Abstract

Nitrogen-doped thiophene plasma polymer [N-ThioPP] thin films were deposited by radio frequency (13.56 MHz) plasma-enhanced chemical vapor deposition method. Thiophene was used as organic precursor (carbon source) with hydrogen gas as the precursor bubbler gas. Additionally, nitrogen gas [N_2_] was used as nitrogen dopant. Furthermore, additional argon was used as a carrier gas. The as-grown polymerized thin films were analyzed using ellipsometry, Fourier-transform infrared [FT-IR] spectroscopy, Raman spectroscopy, and water contact angle measurement. The ellipsometry results showed the refractive index change of the N-ThioPP film. The FT-IR spectra showed that the N-ThioPP films were completely fragmented and polymerized from thiophene.

## Introduction

The existing semiconductor technology lets a silicone material make integrations by a top-down form developed by nano- or molecule technology merged with nanotechnology, biotechniques, and information technology and by a bottom-up method to constitute a device and a circuit with self-alignment of atoms and molecules. Those are common opinions of a majority of experts. In spite of the basic consensus by such experts, the progress in the nano and molecule device research field is very slow, and it is much worse now. There are many causes as possible reasons, but it is recognized that the following are still in question: 'the choice of the stable molecule and design technology,' 'self-alignment technology of atoms and molecules,' and 'technology to form a molecule and contact between the metal electrode for stability' [1,2]. The realization of nanoscale electronics expects the development of bottom-up strategies such as chemical synthesis, self alignment of atoms and molecules, and self-assembled supramolecule. In fabricating the bio-application material, the diamond-like carbon [DLC] films have been a good candidate for some applications such as blood-contacting devices [3] and cell-contacting materials [4] due to their excellent mechanical properties [5-7].

In this work, nitrogen-doped plasma polymer was deposited by nitrogen injection during the plasma-enhanced chemical vapor deposition [PECVD] process without ammonia gas. Also, N-ThioPP thin films were investigated on the surface properties such as surface energy and structural effects.

### Experimental detail

**Figure 1:**
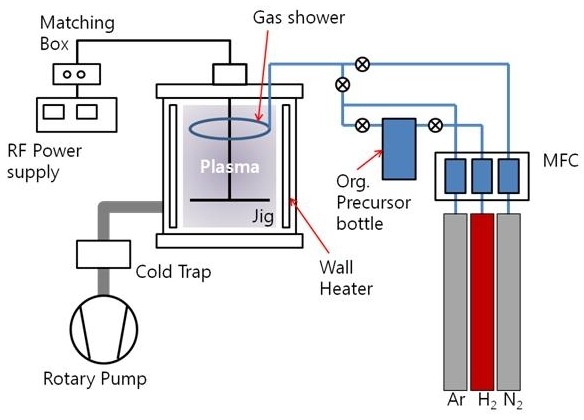
**A schematic diagram of the home-made PECVD system**.

The experiment was carried out in a homemade stainless-steel PECVD system as shown in Figure 1. Silicon(100) wafers were wet-cleaned by sonication with acetone, ethyl alcohol, distilled water, and isopropyl alcohol and dried by N_2 _gas blowing. Also, substrates were dry-cleaned by *in situ *Ar plasma bombardment with 100 W for 15 min. The plasma polymer thin films were deposited by PECVD method. Thiophene was utilized as organic precursor. Thiophene was preheated up to 60°C and bubbled by 50 sccm of hydrogen gas. Additionally, 50 sccm of argon gas was used as a carrier gas. The deposition time was 45 min to make the same thickness at 1 μm. The deposition pressure and temperature were 4.0 × 10^-1 ^Torr and 25°C, respectively. The typical conditions of the PECVD process applied in this study for film deposition are 0, 20, 30, 40, and 50 sccm of N_2 _gas flow.

The chemical bonding type of plasma polymer thin films was investigated by FT-IR spectroscopy (Vertex 70, Bruker Optik Gmbh, Ettlingen, Germany). Moreover, Raman shift of each thin film was investigated by FT-Raman spectroscopy (Vertex 70 with RAM-II, Bruker Optik Gmbh, Ettlingen, Germany). Surface wettability was measured according to water contact angle measurements (Attension, KSV Instruments, Ltd., Helsinki, Finland). The *ex-situ *ellipsometry data of all investigated films were produced by an ellipsometer (GC5A automatic ellipsometry, Gaertner Scientific Corporation, Skokie, IL, USA) at 632 nm to investigate the relationship of film density with the doping amount of nitrogen. Transmittance and bandgap energy of the N-ThioPP thin film were investigated by a UV-Vis spectrophotometer (Optizen 2120UV Plus, Mecasys Co., Ltd., Yuseong-gu, Daejeon, South Korea).

## Results and discussion

**Figure 2:**
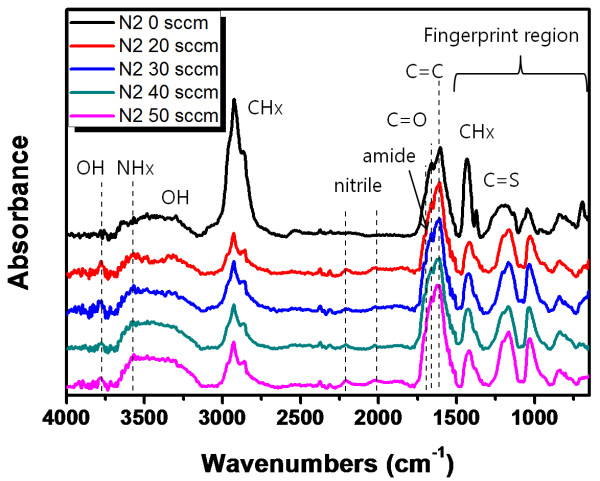
**FT-IR spectra of ThioPP and N-ThioPP with N_2 _flow rate**.

**Figure 3:**
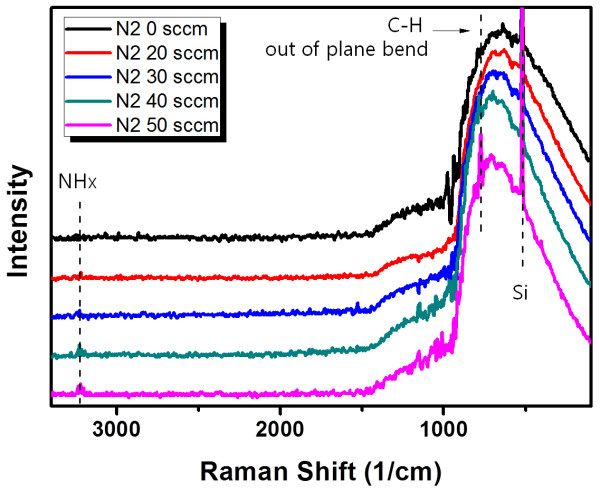
**Raman spectra of ThioPP and N-ThioPP with N_2 _flow rate**.

The bonding state of the plasma-polymerized thin films was analyzed by FT-IR absorption over a range of 4,000 to 600 cm^-1^, as shown in Figure 2. Bands from 700 to 1,050; 1,180; 1,440; 1,601; 1,667; 1,700; 2,025; 2,200; 2,800 to 3,000; 3,200 to 3,600; 3,550; and 3,700 cm^-1 ^corresponded to the alkenes (CH_x_), C = S, CH_x _bending vibrations, C = C; C = O, amide, isonitrile (aromatic), nitrile (aromatic), CH_x _stretching vibrations, OH, NH_x_, and OH bands, respectively [8]. OH absorption bands come from the air during *ex-situ *FT-IR measurement. NH_x _and nitrile absorption band were observed with nitrogen-doped samples. Increasing the N_2 _flow rate during the PECVD led to an increase in the nitrogen doping amounts and nitrogen species binding in the ThioPP thin film. Also, the shape of the fingerprint region is different between the ThioPP and N-ThioPP thin films. Notably, the amide shoulder peak (in the vicinity of 1,700 cm^-1^) is observed only in nitrogen-doped ThioPP samples. From those results, nitrogen was bonded with the ThioPP film by nitrogen injection during the PECVD process. Additionally, Raman spectra also show the same evidence as the IR result. The Raman shift of each sample was shown in Figure 3. The assignments of the bands are observed in the vicinity of 3,200 cm^-1 ^and 770 cm^-1 ^in the NH_x _vibrations and CH out-of-plane bend, respectively [9-11]. NH_x _peaks were increased with increasing N_2 _flow rate. Also, there are no peaks in the vicinity of 1,300 cm^-1 ^and 1,600 cm^-1^. It means that the N-ThioPP thin film is not a DLC film in this experimental condition [12].

**Figure 4:**
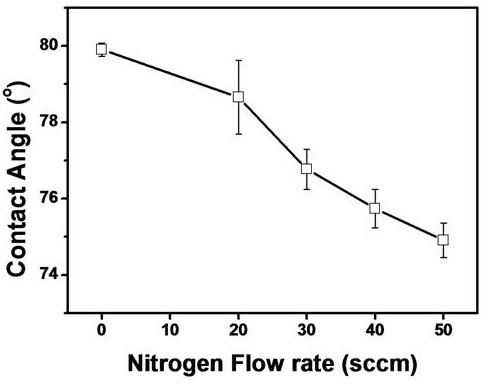
**Water contact angle of ThioPP and N-ThioPP with N_2 _flow rate**.

The change of water contact angles with increasing N_2 _flow rate was shown in Figure 4. The water contact angles were decreased by increasing the N_2 _flow rate. Surface energy of the ThioPP thin film was changed into a more hydrophilic surface by the high flow rate of nitrogen. When 50 sccm of N_2 _gas was inserted during the PECVD process, the water contact angle was 74° which was the lowest value. From the FT-IR spectroscopy, FT-Raman spectroscopy, and contact angle measurements, the chemistry and surface energy of the ThioPP thin film were changed by nitrogen amounts.

**Figure 5:**
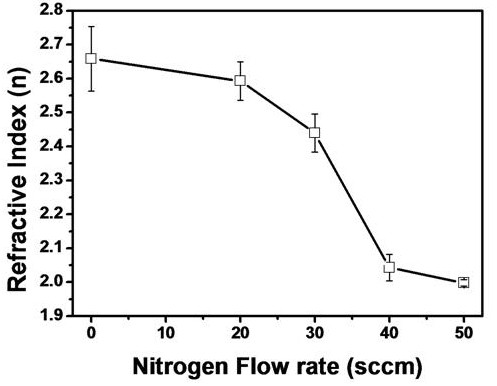
**Refractive indices of ThioPP and N-ThioPP with N_2 _flow rate**.

Figure 5 shows the refractive indices of the plasma polymer thin film with RF power. Refractive index was decreased by increasing the N_2 _flow rate. It means that the density of the plasma polymer thin film was decreased by increasing the N_2 _flow rate due to the disturbance that led to form a high density cross-link between the thiophene molecules in the plasma polymer. Thus, the refractive index of N-ThioPP thin film was decreased by increasing the N_2 _flow rate [13,14].

**Figure 6:**
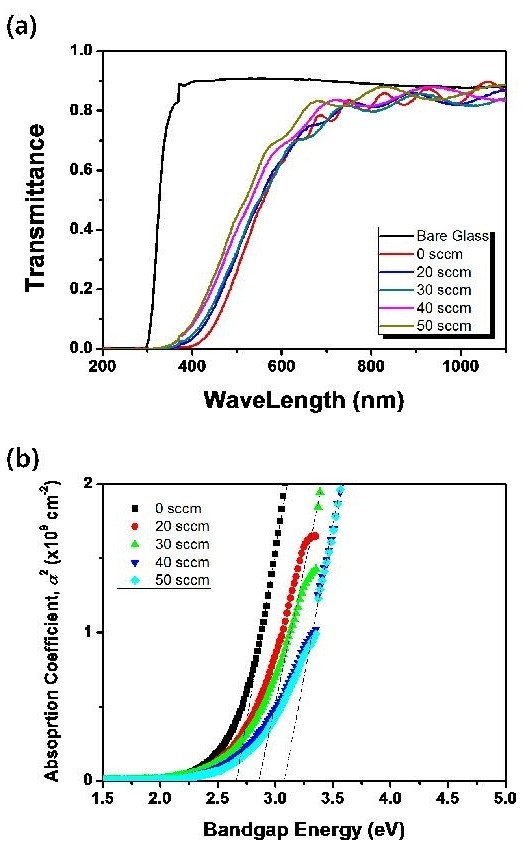
**UV-Vis spectra (a) and bandgap energy (b) of ThioPP and N-ThioPP with N_2 _flow rate**.

We measured that the average transmittance in the infrared range was over 80% for all ThioPP films (in Figure 6a). The optical band gap, *E*_g_, of the thin film could be obtained by plotting *α^2 ^*vs. *hν *(*α *is the absorption coefficient and *hν *is the photon energy) and extrapolating the straight-line portion of this plot to the photon energy axis (Figure 6b) [15-18]. As nitrogen contents increased, the absorption edge shifted to a longer wavelength region (Figure 6a). Figure 6b showed the variation of optical band gap as a function of dopant contents, respectively. Optical band gaps were widened with increasing nitrogen flow rate (in Figure 6b). The reasons were that the densities of electrons were decreased while nitrogen ions were substituted into carbon sites in the films.

## Conclusions

N-ThioPP thin films were deposited on Si(100) by the PECVD method. IR spectra (nitrile band, 2,025 and 2,200 cm^-1^; and the difference of the fingerprint region between ThioPP and N-ThioPP) show that the N-ThioPP thin film was fabricated by nitrogen gas injection during the PECVD process. Moreover, the increasing NH_x _species was definitely shown in the Raman spectra. NH_x _species was increased by increasing the N_2 _flow rate. Also, decreasing the contact angle shows the increasing surface energy of the N-ThioPP thin film with increasing N_2 _flow rate. Additionally, decreasing the contact angle indicates the indirect cause of the increasing nitrogen amounts in the N-ThioPP thin film. Nitrogen atoms bonded with thiophene molecules during the PECVD process. Also, nitrogen disturbs the strong bond between thiophene molecules. Thus, the refractive index of the N-ThioPP thin film was decreased by increasing the nitrogen amount. It indicates that the hardness of the N-ThioPP thin film was controlled by nitrogen amounts in the thin film. UV-Vis spectra of all samples show 80% of transmittance in the infrared region. However, transmittance in the visible region was dramatically changed by increasing the nitrogen amounts. Thus, the energy bandgap of N-ThioPP was increased by increasing the nitrogen amounts.

From those results, nitrogen-doped plasma polymer thin films could be fabricated easily by nitrogen injection during the PECVD process without ammonia as toxic gas. Also, we can control the optical, physical, and chemical properties of the N-ThioPP thin film by controlling of nitrogen flow rate.

## Competing interests

The authors declare that they have no competing interests.

## Authors' contributions

SJC carried out the thin film deposition and analytical studies, participated in the sequence alignment, and drafted the manuscript. JHB participated in its coordination. All authors read and approved the final manuscript.
